# P-1122. Community Transmission and Hospital-Acquired Infections During Outbreaks: Temporal Patterns and Predictive Modeling

**DOI:** 10.1093/ofid/ofaf695.1317

**Published:** 2026-01-11

**Authors:** Jingya Yu, Xianqun Luan, Brian T Fisher, Susan Coffin, David Rubin, Jiasheng Shi, Jing Huang

**Affiliations:** PolicyLab, Children’s Hospital of Philadelphia, Philadelphia, PA; PolicyLab, Children’s Hospital of Philadelphia, Philadelphia, PA; Children’s Hospital of Philadelphia, Philadelphia, Pennsylvania; Children's Hospital of Philadelphia, Philadelphia, Pennsylvania; University of California, California, USA, Philadelphia, Pennsylvania; The Chinese University of Hong Kong, Shenzhen, Shenzhen, Guangdong, China; Department of Biostatistics, Epidemiology, and Informatics, Perelman School of Medicine, The University of Pennsylvania, Philadelphia, Pennsylvania

## Abstract

**Background:**

Hospital-acquired infections (HAIs) threaten patient safety and strain healthcare systems, with heightened risks during outbreaks like COVID-19. While hospital-based prevention is well-studied, the role of community transmission in driving HAIs caused by outbreak pathogens remains underexamined. Understanding this relationship, which may also vary by region and hospital, is critical for preparedness and targeted responses.

**Methods:**

We conducted a retrospective study of 1,814 U.S. acute care hospitals using data of daily COVID-19 HAIs, county-level COVID-19 case counts, ED visits, county and hospital-level characteristics from October 2020 to March 2022. Temporal relationships between community transmission and COVID-19 HAIs were assessed using autocorrelation and cross-correlation analyses. Predictive models were developed using zero-inflated negative binomial (ZINB) regression for daily COVID-19 HAI to address excess zeros and overdispersion. A generalized linear mixed model was developed for high vs. low HAI-to-bed ratios. Model performance was evaluated via time-based validation across key pandemic phases, using rolling train-test splits, and hospital-based validation by repeatedly training on 80% of hospitals and testing on the remaining 20% to assess generalizability across sites.

**Results:**

Community case counts lagged by 9-10 days and ED visits lagged by 13-16 days were strongly associated with HAIs. The ZINB model moderately predicted daily HAI counts, with underperformance during epidemic surges. Meanwhile, the binary classification model for high/low HAI-to-bed ratios achieved strong accuracy (74-91%) and AUCs of 0.81-0.97 across different pandemic phases. Hospital characteristics did not significantly improve model performance.

**Conclusion:**

Community transmission is a leading indicator of HAIs during COVID. Predictive models incorporating lagged community data can support early detection of high-risk periods. Binary risk classification may be more actionable and robust than count-based forecasts, especially during periods of rapid change. These findings support integrating community surveillance into hospital infection prevention and control planning to enhance pandemic preparedness.

**Disclosures:**

Brian T. Fisher, DO, MPH/MSCE, Merck: Grant/Research Support|Pfizer: Grant/Research Support

